# Responses of primate LGN cells to moving stimuli involve a constant background modulation by feedback from area MT^☆^

**DOI:** 10.1016/j.neuroscience.2013.04.055

**Published:** 2013-08-29

**Authors:** H.E. Jones, I.M. Andolina, K.L. Grieve, W. Wang, T.E. Salt, J. Cudeiro, A.M. Sillito

**Affiliations:** a Department of Visual Neuroscience, UCL Institute of Ophthalmology, London, United Kingdom; b Faculty of Life Sciences, University of Manchester, Manchester, United Kingdom; c Department of Medicine – Neurocom and INIBIC (Institute of Biomedical Research of A Coruña), University of A Coruña, Coruña, Spain

**Keywords:** LGN, lateral geniculate nucleus, MT, middle temporal, RF, receptive field, V1, primary visual cortex, lateral geniculate nucleus, feedback influence, area MT

## Abstract

The feedback connections from the cortical middle temporal (MT) motion area, to layer 6 of the primary visual cortex (V1), have the capacity to drive a cascaded feedback influence from the layer 6 cortico-geniculate cells back to the lateral geniculate nucleus (LGN) relay cells. This introduces the possibility of a re-entrant motion signal affecting the relay of the retinal input through the LGN to the visual cortex. The question is whether the response of LGN cells to moving stimuli involves a component derived from this feedback. By producing a reversible focal pharmacological block of the activity of an MT direction column we show the presence of such an influence from MT on the responses of magno, parvo and koniocellular cells in the macaque LGN. The pattern of effect in the LGN reflects the direction bias of the MT location inactivated. This suggests a moving stimulus is captured by iterative interactions in the circuit formed by visual cortical areas and visual thalamus.

## Highlights

• We investigated the influence of area MT feedback on LGN cell responses to visual stimuli.
• We used focal GABA micro-iontophoresis to reversibly block area MT cell responses.
• Inactivating area MT feedback produced clear and reversible changes in LGN cell responses.
• Effects were observed across magno, parvo and koniocellular LGN cell types.

## Graphical abstract

**Figure:**
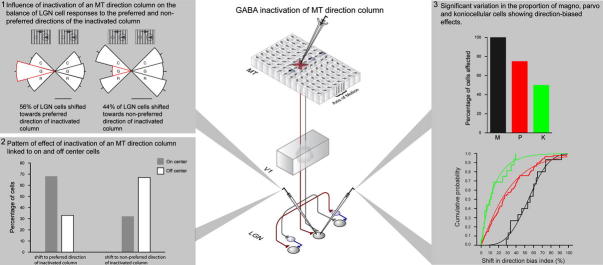

## Introduction

The cortical middle temporal area (MT) is considered to play an integrative role in the processing of visual motion by utilizing the input encoded locally by direction selective cells in the primary visual cortex to generate larger receptive fields that can compute the motion of patterns (Movshon et al., 1985). From this perspective MT has access to information that is unavailable to individual V1 neurons. However one notable feature of the connections linking V1 and MT is that MT provides feedback to V1 (Maunsell and Van Essen, 1983; Shipp and Zeki, 1989) and that this feedback has striking effects on the responses of V1 neurons to flashing and moving stimuli (Hupe et al., 1998). The receptive fields of MT cells are some 10 times larger than those of V1 (Albright and Desimone, 1987) and they provide a feedback projection to a corresponding retinotopic area of V1 (Shipp and Zeki, 1989; Sillito et al., 2006). This feedback includes a projection to layer 6 (Shipp and Zeki, 1989; Rockland and Knutson, 2000) where it has access to layer 6 cortico-geniculate feedback cells and via this route could influence a similar retinotopic area of the LGN. Thus the perspective of the integration carried out in MT is actually available to V1 cells because it clearly influences their responses and potentially available to LGN neurons via the relayed feedback from layer 6. In temporal terms MT feedback has the capacity to influence the early part of the responses of cells in both V1 and the LGN (Hupe et al., 2001; Pascual-Leone and Walsh, 2001; Sillito et al., 2006; Briggs and Usrey, 2007, 2008). Equally it is not immediately apparent what the signature of MT on responses in the LGN might be expected to be.

**Fig. 1:**
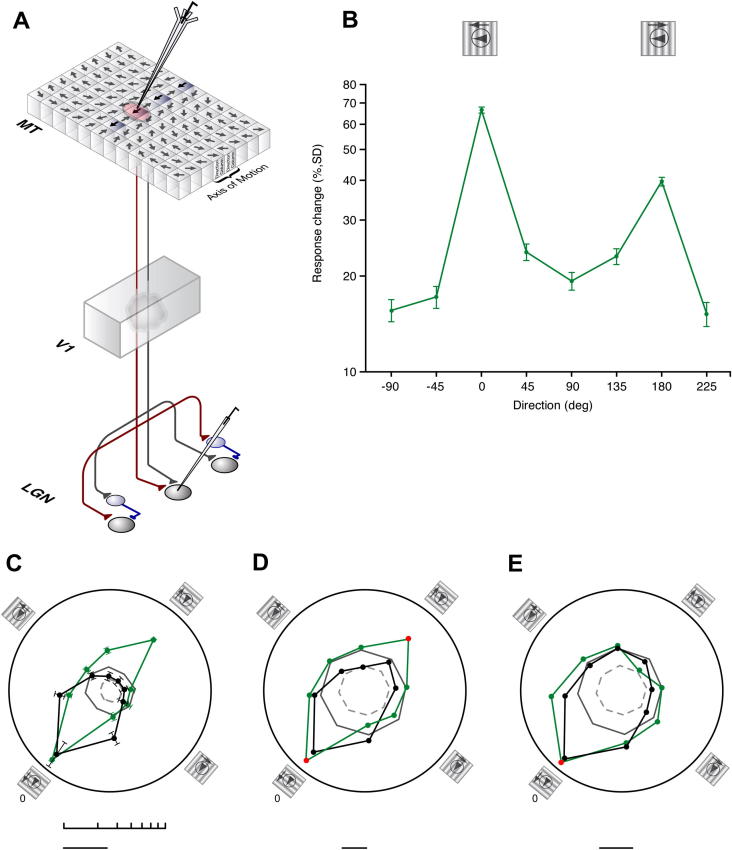
Influence of inactivating an MT direction column on the responses of LGN cells to a range of stimulus motion directions. (A) Schematic summary of feedback projection from MT via V1 to the LGN and experimental method. Drug electrode is shown inserted in a direction column in a schematic of the columnar organization in macaque MT (Albright et al., 1984) and a recording electrode is shown placed in the LGN. (B) Population data summary tuning curve (green line, filled circles) plots the geometric mean of the change in LGN population response magnitude during MT direction column inactivation (*y* axis, logarithmic scale) for a range of test directions (*x* axis). MT column preferred direction 0°. Response changes shown are the geometric mean values for eight LGN cells. Error bars are standard deviations. The iconic representations of stimulus configuration and receptive field are for illustrative purposes only, and not to scale. The circle represents the center of the MT cell receptive field location and the large arrowhead within it denotes the preferred direction of the MT column. The smaller arrowheads indicate the direction of stimulus motion. (C) Population data summary polar tuning curve (green line) plots, on a logarithmic scale, the geometric mean of the change in LGN population response magnitude during MT direction column inactivation for a range of test directions. Error bars are standard deviations. Logarithmic scale bar = 10–80%. The black tuning curve plots, on a linear scale, the mean averaged control direction tuning curve for the associated MT cell population data prior to inactivation (error bars denote 1 SE). MT column preferred direction 0°. Linear scale bar = 20 spikes/s (s/s). Iconic representations of stimulus configurations follow same conventions as in (B). (D, E) Two representative examples of the effect of MT inactivation on individual LGN cell tuning curves. In each case, the green curve plots the percentage change in LGN response magnitude during MT direction column inactivation. Responses during MT inactivation that differed significantly to control levels (see Experimental procedures) are marked by red filled symbols; non-significant changes are denoted by green symbols. The solid black and gray lines plot the control responses of the MT and LGN cell respectively. For (D), scale bar equates to: green curve 20%, black curve 10 s/s, gray curve 85 s/s. For (E), scale bar equates to: green curve 20%, black curve 6 s/s, gray curve 50 s/s. Light gray dashed circle marks 0 s/s.

In this paper we describe a method that reveals an influence from MT on LGN cell responses. It builds on the fact that in macaque MT there is a columnar organization for direction (Albright et al., 1984; Born and Bradley, 2005) with a broadly smooth progression of shifts in directional preference side by side with corresponding columns tuned to the opposite direction (Fig. 1A). We took advantage of this arrangement to selectively and reversibly pharmacologically manipulate the output from cells in an MT direction column during visual stimulation and checked for the effect of this manipulation on the responses of LGN cells driven by the same visual stimulus. A key point here is that because most LGN cells are not notably directionally selective (but note Lee et al., 1979; Xu et al., 2002) we assumed that if they received a feedback influence from MT it would normally pool the output from a complete subset of direction columns. Thus our pharmacological manipulation of an MT direction column, if effective would, in changing the strength of the feedback from that column, reveal an asymmetry in the directional response of LGN cells receiving the feedback that was linked to the preferred direction of the MT column. This is in fact what we observed.

## Experimental procedures

### Anesthesia

Experiments were carried out on anesthetized [sufentanil (4–8 μg/kg/h in a glucose enriched Hartmann’s solution) and a mixture of 70% N_2_O and 30% O_2_ supplemented, as required, with halothane (0.1–0.5%)] and paralyzed (0.1 mg/kg/h vecuronium bromide) female primates (*Macaca mulatta*) as previously described (Jones et al., 2001). All procedures were in accordance with British Home Office licence requirements and approved by the local ethical review committee at UCL’s Institute of Ophthalmology.

### Recording and iontophoretic methods

**Fig. 2:**
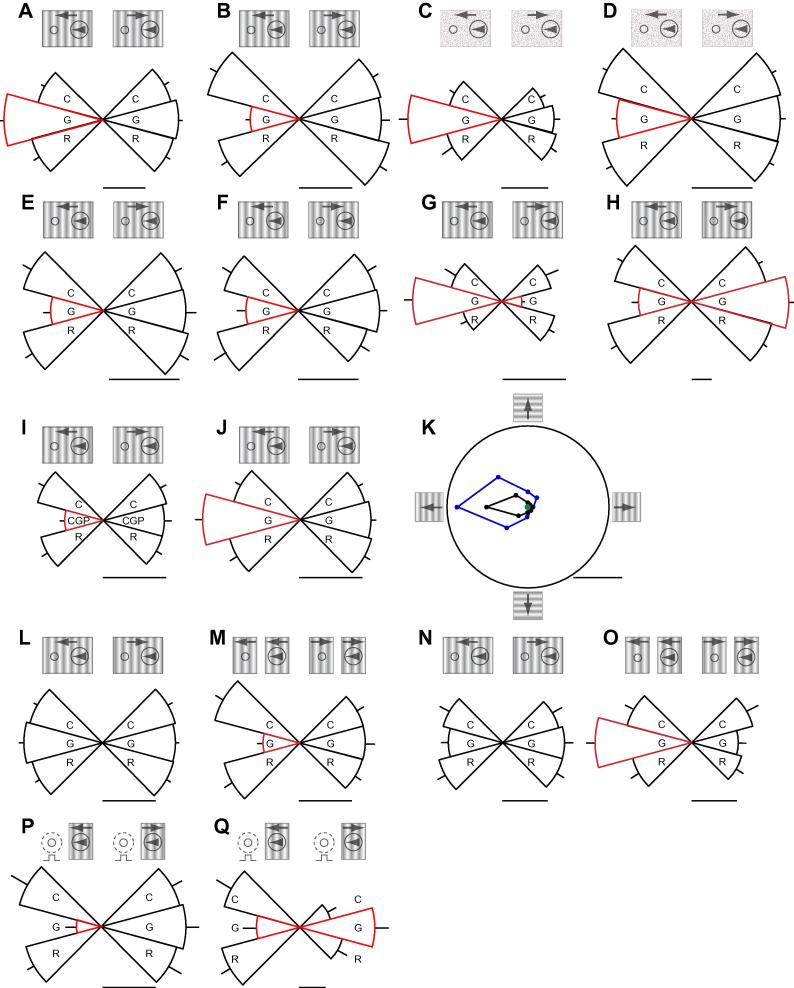
Inactivation of an MT direction column can evoke clear and reversible changes in the responses of LGN cells. (A, B) The histograms document the responses of two typical LGN cell examples to a large patch of grating drifting in the preferred (left) and non-preferred (right) directions of the MT inactivation site, before (C), during (G) and after cessation of (R) GABA application in MT. Red shading indicates that there was a significant difference (*P* < 0.05, paired *t*-test, see Experimental procedures) between the responses observed during control and MT inactivation conditions. Error bars 1 SE. Scale bars = 10 s/s. Here, and in all subsequent panels and figures, the iconic representation of the MT and LGN cell receptive field locations and stimulus configuration drawn above the records are for illustrative purposes only, and not to scale. (C, D) The histograms document the responses of two typical LGN cell examples to a large patch of texture drifting in the preferred (left) and non-preferred (right) directions of the MT inactivation sites before (C), during (G) and after cessation of (R) iontophoretic application of GABA in MT. Error bars 1 SE. Scale bars = 20 s/s. (E, F) Repeatability of influence of MT inactivation over time. The histograms document the responses of an LGN cell to a patch of grating drifting in the preferred (left) and opposite directions of motion (right) of the MT inactivation site, before (C), during (G) and after cessation of (R) GABA application in MT. The histogram in (E) shows the responses during the initial application of GABA in MT and that in (F) the responses observed during a subsequent test cycle recorded over an hour later. Scale bar = 10 s/s. (G, H) Reciprocal effects of inactivation of an MT direction column on LGN cell responses to the MT site preferred direction of motion and its reverse. The histograms document the responses of two LGN cells to a patch of grating drifting in the preferred (left) and opposite directions of motion (right) of the MT inactivation site, before (C), during (G) and after cessation of (R) GABA application in MT. Scale bar = 15 s/s. (I–K) Iontophoretic application of GABA and the GABA B receptor antagonist CGP 55845 in MT evoke opposite effects on the responses of LGN cells. The histograms in (I) document the responses of an LGN cell to a patch of grating drifting in the preferred (left) and opposite directions of motion (right) of the MT locus, before (C), during (CGP) and after cessation of (R) CGP 55845 application in MT. The histograms in (J) shows the responses of the same cell observed during GABA application in MT. Scale bar = 15 s/s. During CGP application in MT, the LGN cell’s response to the stimulus moving in the MT preferred direction of motion was significantly reduced whereas it was significantly increased during GABA iontophoresis in MT. This reciprocal pattern of effect held across our sample (*n* = 5) of cells tested with both GABA and CGP 55845, with a significant negative correlation in the normalized response change observed in the two conditions (*R* = −0.9, *P *= 0.037, Spearman rank order correlation test). The polar plots in (K) document the responses (in s/s) of the MT locus to a large patch of drifting grating presented at a range of test directions before (black) and during (blue) local micro-iontophoretic application of CGP 55845. There was no response during local iontophoretic application of GABA (green). Scale bar = 80 s/s. (L–O) Differential effects of inactivation of an MT direction column on LGN cell responses to single and paired stimulus configurations. The histograms in (L) document the responses of an LGN cell to a large, single patch of grating drifting in the preferred (left) and opposite directions of motion of the MT inactivation site (right), before (C), during (G) and after cessation of (R) GABA application in MT. Scale bar = 15 s/s. The histograms in (M) document the responses of the same LGN cell in the presence of two patches of grating, located over the MT and LGN cell receptive fields respectively before (C), during (G) and after cessation of (R) GABA application in MT. Both patches drifted either in the preferred (left) or opposite (right) directions of motion of the MT inactivation site. Scale bar = 15 s/s. The histograms in (N, O) document the response of another LGN cell, before (C), during (G) and after cessation of (R) GABA application in MT to a large, single patch of grating (N) and to two patches of grating (O) drifting in the preferred and non-preferred directions of motion of the MT inactivation locus. Scale bars = 15 s/s. (P, Q). Inactivation of an MT direction column evokes clear and reversible changes in LGN cell responses to flashing stimuli. The histograms document the responses of two LGN cells before (C), during (G) and after cessation of (R) GABA application in MT. The stimulus located over the LGN cell RF comprised a stationary flashing spot while the stimulus located over the MT cell RF comprised a patch of grating, drifting in either the preferred (left) or non-preferred (right) directions of motion of the MT inactivation site. Scale bars = 10 s/s. Error bars 1 SE.

We recorded single unit extracellular responses from LGN cells using multi-electrode arrays of tungsten in glass electrodes and also recorded extracellular responses from single units in area MT using a tungsten-recording electrode glued to a five barrelled glass iontophoretic pipette (Wang et al., 2006). In preliminary tests, we determined the receptive field location of all cells using a battery of tests, and the preferred direction of motion of the MT cell (Jones et al., 2001, 2002; Wang et al., 2006). LGN cells were classed as magnocellular, koniocellular and parvocellular based on physiological response properties, electrode depth and stereotypical shifts in eye preference and subsequent histological reconstruction of electrode tracks (Wiesel and Hubel, 1966; Jones et al., 2012; McAlonan et al., 2008). We then used focal micro-iontophoretic drug application of GABA (0.5 M, pH 3, ejection current was adjusted to suppress the visual response of the MT cell to its preferred direction and ranged from 20 to 60 nA, through one or two barrels, mean 40 nA) to reversibly inactivate the responses of the MT direction column (Fig. 1A) and explored the effects of this on the responses of the LGN cells to patches of drifting gratings moving in the MT column’s preferred direction of motion and its reverse. In all cases, we compared the effects of GABA micro-iontophoretic application in MT on the LGN cell responses to a stimulus drifting in the MT cell’s preferred direction and its reverse. Additionally, in some cases, we incorporated a wider range of stimulus motion directions into our testing protocol to encompass 360° in 45° steps and presented in a randomized, interleaved sequence. In a small number of cases, we compared the effect of focal micro-iontophoretic drug application of the GABA_B_ receptor antagonist CGP 55845 (Brugger et al., 1993; Wang et al., 2006) (2.5 mM in 150 mM NaCl, pH 3.5) which reversibly enhanced the visual responses of the MT direction column (Fig. 2K) on the LGN cell responses. Visual stimuli comprised either a large patch of sinusoidal grating positioned such that the stimulus encompassed the receptive fields of both cells, or two discrete stimulus patches positioned over each individual receptive field location. In the latter case, the stimulus located over the MT cell always comprised a patch of drifting sinusoidal grating. The LGN stimulus either comprised a patch of drifting sinusoidal grating (of the same spatial frequency and drift rate used for the MT cell stimulus patch) or a stationary flashing spot of light. The spatial frequency was normally 2 cpd (range 1–3), temporal frequency 3 Hz (range 2–4), contrast 0.36 (defined as (*L*_max_ − *L*_min_)/(*L*_max_ + *L*_min_)) and mean luminance 50 cd m^−2^. Typically we presented two to five stimulus cycles of each stimulus condition, repeated over 5–25 trials. We also checked the effect of inactivating an MT direction column on the responses of 23 LGN cells to drifting texture and observed similar results to the tests with grating stimuli (15 (65%) of cells tested with texture showed the same pattern of significant shift in their directional bias as observed for the cells tested with sinusoidal gratings).

### Data analysis

Responses were computed from the mean firing rate averaged over the full duration of the stimulus presentation. We compared responses before, during and after focal MT inactivation. Responses of LGN cells were only included for analysis if the responses of the simultaneously recorded MT cell were suppressed during GABA application. LGN cells were only considered to show a change in response magnitude if there was a significant difference between response levels evoked during control and MT drug application conditions (*P *< 0.05, paired two tailed *t*-test) and if the response levels recovered following cessation of drug application. For both control and MT inactivation conditions, we looked to see if there was a significant difference (*P *< 0.05, paired two tailed *t*-test) between the LGN cell’s responses to the two directions of stimulus motion and regarded cells as showing a significant change in direction bias if the cell showed differential effects (i.e. no significant response bias for one condition and a significant bias to the other or if the cell showed significant but opposite polarity biases during the two conditions). In order to quantify the degree of shift in direction bias during MT inactivation, we first independently determined a direction index for control and MT inactivated conditions according to the standard formula DI = (1 − *R*_min_/*R*_max_) * 100 where *R*_max_ is the response to the direction of motion evoking the largest response and *R*_min_ is the response to the reverse direction (following subtraction of spontaneous activity levels) thus 0 would indicate no direction bias and 100 totally direction selective responses. For cells where the maximal responses for control and drug conditions were evoked by the same direction of motion, we subtracted the smallest DI from the largest to obtain the net change in direction bias index, whereas for cells where maximal responses for control and drug conditions were evoked by opposite direction stimuli, we added the two DIs together to obtain the net change in bias index. We used the same methodology to calculate a control-recovery index. We also computed responses from the F1 response component (Enroth-Cugell and Robson, 1966) for grating stimuli and processed the data as described above. This revealed the same pattern and strength of effect with the proviso that in 5% of our sample we observed directional bias effects using the F0 component (mean) but not for the F1 component. Unless otherwise indicated, for population statistics we used the geometric mean as a measure of central tendency. Non-parametric tests were used for population data comparisons as the data were not normally distributed.

### Histology

At the end of each electrode penetration, small electrolytic lesions (3–5 μA for 3–5 s, electrode negative) were made at key recording sites along each electrode track to enable subsequent histological verification of the recording locations (Jones et al., 2001). We confirmed that our electrodes were located in MT using conventional histological criteria (Gallyas, 1979; Van Essen et al., 1981).

## Results

**Fig. 3:**
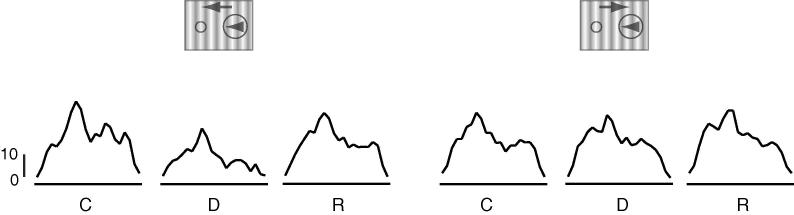
Example documenting the effect of inactivation of an MT direction column on the responses of an LGN cell plotted as peri-stimulus time histograms (PSTHs). The wrapped PSTHs document the responses of the cell illustrated in Fig. 2B. Responses to the preferred (left) and non-preferred (right) directions of motion of the MT inactivation site are shown before (C), during (D) and following recovery from (R) GABA application in MT. Scale bars = 10 s/s. Fifteen milliseconds bins, traces smoothed with a Gaussian window at 1.5 times the bin width.

Our experimental approach (Fig. 1A) involved testing the visual responses of LGN cells to patches of drifting grating or texture (Figs. 1–3) before, during and after the focal micro-iontophoretic application of GABA to selectively block the activity in an MT direction column (see Experimental procedures). Effects on the MT column were assessed from the quantified responses of a single isolated recording of an MT cell in the column (e.g. Fig. 1) and by listening to the background multi unit activity. Using stimuli that encompassed the LGN fields and that of the MT column, the starting point in all our tests was to ascertain whether there was an effect on the responses of LGN cells to the stimulus moving in the MT column’s preferred direction.

### Nature and specificity of effects

Our basic observation was that inactivation of an MT direction column produced clear and reversible changes (mean 33.7%, 95% confidence interval 28.18–40.46, *n* = 126) in the responses of the majority (116/126) of LGN cells tested to the stimulus. This highlighted the fact that the control responses of LGN cells to moving stimuli of this type include a significant influence from feedback originating in MT. We then carried out a series of tests to explore the specificity of the effects in relation to the direction bias of the inactivated MT column. Where we had exceptional stability at both the location in MT and the LGN recording sites we constructed complete directional tuning curves before, during and after drug application. The LGN cells all had receptive fields that were encompassed by the field of the cell recorded in MT. Under control conditions the LGN cells responded more or less equally to all directions of the test stimulus. During the inactivation of the MT direction column there was a systematic change in these responses. This change was focused on the axis of the preferred direction of the MT column and on the reverse direction along this vector (Fig. 1) with the largest effects in the preferred direction. We plot the geometric mean of the change in response magnitude for this group of LGN cells (*n* = 8) during the inactivation of the MT direction column (Fig. 1B, C) and the population average control tuning curve for the MT cells (Fig. 1C) taken prior to inactivation (there was no response during inactivation) in Fig. 1, and give examples of individual LGN cells and the associated MT cell(s) in Fig. 1D, E. The geometric mean values for the change in LGN cell response magnitude during MT direction column inactivation (Fig. 1B) showed a significant difference across the directions as a whole (Friedman ANOVA, *P *< 0.001) and a significantly greater effect on the response to the MT column preferred direction (*P *< 0.01, Conover test) than all other directions bar the reverse direction. These data show that our inactivation procedure produced a specific pattern of influence linked to the preferred direction of the inactivated MT column and its reverse direction. Although the effects at the preferred direction of the MT column were larger, the bidirectional nature of the effect in the LGN however contrasts with the unidirectional preference of the control MT cell responses (Fig. 1C). It would be compatible with the view that MT direction columns are paired with those representing the reverse direction at the same location (Albright et al., 1984; Born and Bradley, 2005) and imply a degree of interlinking in the connectivity between these, such that our drug applications influenced both mechanisms. However, the absence of significant effects on the directions outside this preferred vector, indicate that our drug effects were selective to the response domain vector of the column containing the recorded MT cell. It is crucial to underline here that we are showing that removal of the influence from an MT direction column results in a directionally asymmetric response in LGN cells to stimuli moving in the preferred versus the non-preferred direction of that MT direction column. In this sense the lack of significant directional bias in the control responses of the LGN cells would reflect a balanced feedback from MT in the direction domain, but this feedback would nonetheless be modulating their responses to the moving stimuli.

### Distribution of effects in preferred and null directions of the inactivated MT column

Whilst the observed effects of inactivation of an MT direction column were tuned to the response domain vector of the inactivated MT column we sought to check whether we could obtain more selective effects restricted to the preferred direction only by reducing the drug spread. To this end we checked the effects on the LGN cell responses to the preferred and null directions of the inactivated MT columns in detail across our sample of cells in a series of tests where we only checked responses to these two directions (*n* = 118) and were thus able to restrict the potential for drug spread, enabling rapid recovery and repeat tests. Brief inactivation of the MT direction column resulted in a significant change (see Experimental procedures) in the balance of the response of 75% (88/118) of the LGN cells tested to the MT preferred and non-preferred directions of motion. Of these just over half (56%) showed a shift to the preferred direction (e.g. Fig. 2A, C) and the remainder (44%) to the non-preferred direction (e.g. Fig. 2B, D). These opposite direction changes followed from the fact that in the one set of cases the responses in the preferred direction were enhanced during MT inactivation (e.g. Fig. 2A, C) and in the other reduced (e.g. Fig. 2B, D). An immediate question is whether these differences reflected drug spread or the location of the drug electrode with respect to the boundaries of the MT direction column. In all cases the drug application was as brief as possible to just cover the test of the responses to the two directions. The brief application of GABA in MT stopped the recorded cells firing but the recovery of the response was immediate when the drug was switched off. The effects could be repeated without change in observation (Fig. 2E, F) so there was not a time variant influence. Possibly the most cogent evidence against the fact that the differences reflected drug spread, preparation state, or variations of electrode location in the boundaries of the MT direction column is that we observed opposite direction effects in simultaneously recorded LGN cells. Thus one location in MT, and one brief period of drug application stopping firing in the MT direction column, enhanced the MT preferred direction response of one simultaneously recorded LGN cell and reduced it for another with full recovery after (e.g. Fig. 2A, B and M, O). This held for approximately two thirds (65%) of our simultaneously recorded cell groups. This suggests that the variation in effect follows from a distinction in the way the feedback influenced circuitry in the LGN. There was no correlation in the pattern of effect to the relative location of the LGN fields to the MT field, eccentricity or subdivision into magno, parvo and koniocellular streams. However we found that during MT inactivation, 68% of “on” center cells (36/53) showed a shift to the preferred direction of the inactivated column and 32% shift to the opposite direction, whilst 67% of “off” center (18/27) showed a shift to the opposite direction and only 33% showed a shift to the preferred direction. These proportions are significantly different at *P *= 0.0018 Chi square. This further supports the idea that the different directions of effect reflect some specific functional feature of the micro-organization of the feedback.

If these effects reflected a robust feature of the circuitry one would predict that enhancing the response level of the MT direction column as opposed to inhibiting it would produce an opposite direction effect. To examine this we prepared a drug electrode that contained both GABA and the GABA B receptor antagonist CGP 55845. We show an example illustrating the predicted opposite direction effects in Fig. 2I, J. Iontophoretic application of the GABA B receptor antagonist CGP which increased MT cell responses without changing response specificity (see Fig. 2K) resulted in a decrease in the LGN cell’s response to the preferred direction (Fig. 2I) whilst application of GABA which blocked MT cell responses resulted in an increase in the LGN cell’s response to the preferred direction (Fig. 2J). We repeated this observation on five cells to the same effect but did not routinely use CGP in the main body of the experiments because longer time courses for the recovery from CGP made repeated testing difficult. These data suggest a clear and direct link between the pharmacological manipulation and the pattern of effect. They underline the view that the pattern follows from the discrete organization of the circuitry that enables two different polarities of effect. Indeed in 27/88 cells, GABA application in the MT direction column produced reciprocal changes in the two directions of motion (Fig. 2G, H), i.e. the response to the preferred direction went up and that to the reverse direction went down (17) and vice versa (10). Showing in effect the two patterns in the same cell but coupled to the preferred and null directions of the MT column inactivated. We would highlight the point that in the organization of MT direction columns (Albright et al., 1984; Born and Bradley, 2005) the back-to-back tiling of the sequence of direction shift involves adjacent opposite direction pairs. This feature of the neuronal architecture suggests a very specific reciprocal pairing of opposite direction effects and our data may simply reflect this mechanism at the level of MT.

Across our sample as a whole (*n* = 118), the mean shift in direction bias from control to drug run was 18.3% (CI 13.36–25.02) during brief inactivation of the MT direction column but this value was considerably higher if one considered just the subgroup of LGN cells (*n* = 88) that showed a significant change (see Experimental procedures) in the balance of the response to the MT preferred and non-preferred directions of motion during MT inactivation. For this latter group, the mean shift in direction bias from control to drug run was 35.6% (CI 31.40–40.27). This compared to a shift of only 6.6% (CI 5.05–8.73) between control and recovery recordings.

### Spatial summation across columns

A feature of the columnar organization of early visual cortical areas is that there is a partially shifted overlap of the representation of visual space from one complete subset of columns representing the feature space mapped by the cortical area to the next complete subset mapping an adjacent area. Thus the direction of motion represented by the column inactivated by our electrode would also be partially covered by a column tuned to the same direction and driven by an adjacent but partially overlapping area of visual space. This would repeat in the “tiled representation” of visual space across the repeating subsets of direction columns. Thus our inactivation would only produce a small reduction in the feedback from MT driven by our stimulus for the direction of motion represented by the specific column affected by our electrode. However we reasoned that if rather than use a single stimulus we removed a section of the visual stimulus between the MT field and the LGN fields we would reduce the feedback from columns tuned to the same direction of motion in the tiled representation. If this were the case then with such a bipartite stimulus the inactivation of the column sampled by our drug electrode should reveal a larger effect than seen with a single stimulus because there would less compensatory influence from columns sampling the intervening visual space. This is what we observed. We show examples of two simultaneously recorded LGN cells that when tested with a single drifting grating showed no significant change in response during inactivation of the MT direction column (Fig. 2L, N) but a clear and significant effect of the inactivation when tested with a bipartite stimulus formed by removing the central section of the single stimulus (Fig. 2M, O). Overall for the sample of cells we tested with single and bipartite stimuli (*n* = 21) significantly (*P *= 0.002098 Wilcoxon Matched Pairs test) larger changes in direction bias occurred during inactivation of an MT direction column if we removed the central section of the single stimulus (average gap size 1.8° ± 0.26 SE) to create two stimuli (geometric mean 45.8%, 95% CI 36.71, 57.20) as opposed to one (23.9%, 95% CI 15.30–37.43). This is consistent with the interpretation that stimulation of MT direction columns covering intermediate retinotopic locations between the inactivated column and the LGN cells mitigates the effect of the inactivation.

It is also pertinent to note that the introduction of the gap would sometimes change the balance of the control responses revealing a larger response to one direction than the other. In the absence of any bias in the response to a single stimulus this could be argued to be a context dependent modulation of the LGN cell responses (Fig. 2M). One might consider that it reflected the role of subcortical mechanisms, but it is salient that inactivation of the MT direction column reversed the effect (see also Fig. 2Q below). This suggests the difference in the response to the two directions of motion for these bipartite pairs derived from the interplay of the two stimuli in the integration carried out by circuitry at the level of MT and involved the direction column inactivated by our drug.

### Effects of feedback from MT on the response of LGN cells to flashing stimuli

Another question arising from these observations is whether a necessary condition of the influence of MT on the LGN cells was the relative motion context over the MT and LGN cell receptive fields. To examine this we examined the effect of inactivation of an MT direction column on the responses of LGN cells to a stationary flashing stimulus (Fig. 2P, Q). In this case, as before, the MT column was driven by a stimulus alternately drifting in the preferred and non preferred directions and the LGN cells by a stationary flashing stimulus. The simple observation was that during the inactivation of the MT direction column the response to the flashing stimulus was differentially changed (Fig. 2P, Q). For this group the effect was seen in 14 out of 16 cells tested (87.5%) and the mean shift in direction bias was 47.5% (geometric mean, 95% CI 35.1–64.4). As for the moving stimuli we observed examples of selective response reduction, selective response enhancement and a reciprocal influence (Fig. 2P, Q). Again for the reciprocal effects the net shift in response was synergistically selective in the directional domain. With this stimulus paradigm we also observed examples (Fig. 2Q) where, as for the bipartite moving stimuli (Fig. 2M), the stimulus context under control conditions induced an asymmetric response of the LGN cells and this was reversed during the inactivation of the MT column. It is also important to reiterate that, as these records show, the effects of the MT inactivation immediately returned to the control situation in the recovery records.

### Variation of feedback effects with LGN cell type and separation of LGN and MT cell receptive fields

**Fig. 4:**
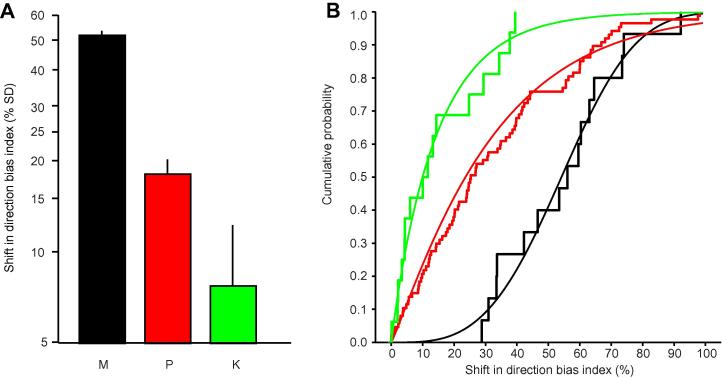
Summary of the effects of MT direction column inactivation across our LGN cell sample. (A) Histogram plots the geometric mean values, on a logarithmic scale, for the shift in direction bias index across the population of magno (M), parvo (P) and koniocellular (K) cells tested. Error bars 1 SD. (B) Cumulative probability distribution (cpd) of the magnitude of the change in direction bias index (%) for magno (black), parvo (red) and koniocellular (green) cells. For each cell class, the thicker line plots the actual cpd of the data whereas the thinner line shows the best non-parametric fit to the data.

We examined whether there was any variation in the effect of MT inactivation across the three types of LGN cells (Fig. 4A, B). The geometric mean of the change in directional bias was 51.2% for magnocellular cells (*n* = 15, 95% CI 42.08–62.28), 17.9% for parvocellular cells (*n* = 87, 95% CI 12.19–26.41) and 7.7% for koniocellular cells (*n* = 16, 95% CI 3.45–17.20). These values across the three cell classes were significantly different to each other (*P *= 0.0001, Kruskal–Wallis ANOVA). There was also a significant variation in the proportion of cells showing direction-biased effects during MT inactivation within the three cell groups (*P *= 0.00342, Freeman–Halton extension of the Fisher Exact Probability Test). All magnocellular cells (*n* = 15) showed this effect compared to approximately 75% of parvocellular cells (65 out of 87 cells tested) and 50% of koniocellular cells (8 out of 16 cells). Given this variation, we also examined the magnitude of effects observed for only those parvo and koniocellular cells that showed a significant direction biased effect (see Experimental procedures). The mean values were 34.7% (95% CI 29.96–40.10) and 22.1% (95% CI 13.83–35.39) for parvocellular and koniocellular cells respectively. Again, these values were significantly different across the three cell classes (*P *= 0.00453, Kruskal–Wallis ANOVA).

We also checked if the magnitude of effect was influenced by the distance (measured in both degrees and in units of MT receptive field (RF) radius) between the LGN and MT field centers or not. Although there was no influence for konio and magnocellular cells (*P *> 0.05, Spearman rank order correlation test for both cell groups) there was a weak but significant correlation for parvocellular cells (*R *= 0.37, *P *= 0.0004 and *R *= 0.24, *P *= 0.027 for distance in degrees and units of MT RF radius respectively).

## Discussion

Our experiments probed the question of whether the potential for cascaded feedback from MT to the LGN via connections to layer 6 of V1 is realized by an MT influence on LGN cell responses. They show the presence of this influence. By briefly inactivating an MT direction column we revealed reversible changes in LGN cell responses that were linked to the axis of the preferred direction of the MT column inactivated. The implication is that the responses of LGN cells to moving stimuli are significantly influenced by feedback originating in MT. This may seem counterintuitive because LGN cell responses in primate are not considered to be in general directionally selective although they do exhibit response asymmetries (Lee et al., 1979; Xu et al., 2002). However we revealed this by removing the influence of a direction column from the control situation and thus introducing an asymmetry in the feedback. Under control conditions, with a simple moving stimulus, the responses of an LGN cell to the stimulus would be more or less equally balanced in all directions because the feedback influence from MT would also be equally balanced. Basically it seems LGN cell responses are modulated by MT feedback that introduces the context of salient motion integrated over a much wider area than the subcortical process in isolation. Because the changes in the LGN cells responses change the input to V1, and in turn the input to MT with consequential change in MT responses and its feedback back to V1 and the LGN, our data argue for a constant reiterative mechanism locking onto the stimulus. From this perspective the representation of the stimulus sits in the interaction between the levels and is best conceived in terms of a dynamic interplay between top down priors reflecting the best fit of the input to the higher level abstraction and the circuitry parsing the ascending information. Issues that bear on this interpretation of the way the system works are clearly set out in recent reviews (Sillito et al., 2006; Gilbert and Sigman, 2007). In particular, it is cogent to note that feedback down the neuraxis from MT is fast and will engage the circuitry in V1 and the LGN in a way that reflects the characteristics of the stimulus driving MT and that top-down influences will be updated dynamically.

### Distribution and specificity of effects in preferred and null directions of the inactivated MT column

It is important to emphasize that the changes we observed always led to a change in direction bias to the directional vector defined by the MT column inactivated and in some cases involved reciprocal changes to the responses to the preferred and non-preferred directions of the column inactivated (e.g. Fig. 2G, H, Q). A crucial point is that we highlighted the effect following from inactivating a particular focus in the map of direction columns, and used this to gain an insight into the functional organization of the feedback from MT as a whole as illustrated in Fig. 1. This bias in effect reflects what one would predict from the organization of direction columns in MT (Fig. 1A) and whilst small effects on immediately adjacent direction columns (with similar and overlapping direction tuning curves) cannot be excluded the selectivity of our manipulation is clear. This also needs to be placed in the perspective of the direction tuning curves of MT cells themselves. The effect is possibly as sharp as one could predict it to be given the manipulation we carried out, the pattern of organization of MT direction columns and the tuning width of MT cells. The key finding is that this feedback does exert a significant influence on LGN cell responses. The global feedback from MT will of course involve the integration of the influence from a complete subset of direction columns. Our results in effect disclose new properties of the normally invisible but always ongoing reiteration in the feedback–feedforward circuitries between visual cortical areas and visual thalamus. From this perspective, the seemingly balanced subcortical influence of the higher motion area in response to grating stimuli will shift out of balance when viewing natural scenes with adjacent stationary and moving areas, possibly increasing contrast and quality of perception.

### Spatial summation across columns

A further point we considered was the effect of a partially shifted overlap of the representation of visual space from one complete subset of MT columns representing the feature space mapped by the cortical area to the next complete subset mapping an adjacent area. The direction of motion represented by the column inactivated by our electrode would also be partially covered by a column tuned to the same direction and driven by an adjacent but partially overlapping area of visual space. Thus in the “tiled representation” of visual space across the repeating subsets of direction columns our inactivation of a single column would be offset by the influence of the next subsets. Where we were recording from LGN cells with non overlapping receptive fields we reasoned that if we introduced a gap into our stimulus in the portion of visual space lying between the MT and LGN cell fields this would reduce the strength of the drive to the tiled representation of MT columns influencing the LGN and by this token the effect of our inactivation should be stronger because it would be less masked by these supplemental feedback influences. This indeed was the case and is consistent with the view that the LGN receives a tiled convergent feedback from repeating subsets of MT direction columns.

### Variation of feedback effects with LGN cell type

An interesting feature of our data is that some LGN cells showed a shift in bias towards the preferred direction of the inactivated MT column and others a shift to the null direction. We observed opposite direction shifts in simultaneously recorded LGN cells and so these effects would seem to reflect some facet of the organization of the circuitry engaged by the stimulus. The fact that we saw a significant difference in the pattern between “on” and “off” center cells, where the majority (68%) of “on” center cells showed a shift towards the preferred direction of the inactivated MT column whilst the majority (67%) of “off” center cells showed a shift away, underlines this point (see results). It is pertinent that there is both evidence suggesting that feedback from layer 6 simple cells to the LGN is phase specific (Wang et al., 2006) and thus capable of differential effects on “on” and “off” center LGN cells and that many of the feedback cells are directionally specific (Grieve and Sillito, 1995; Briggs and Usrey, 2009). These points serve to highlight the capacity of the neural organization to deliver the two patterns of effect and raise the possibility of a tiled phasing of the influence in relation to the predicted sequence of contrast borders.

There is a basis for very fast feedback from V1 to the LGN in the primate (Sillito et al., 2006; Briggs and Usrey, 2007, 2009) and whilst the bias to magnocellular effects (100%) is entirely predictable from what is known of the system (Van Essen and Maunsell, 1983; Maunsell et al., 1990) there are strong grounds for expecting effects on parvo and koniocellular cells (Raiguel et al., 1989; Shipp and Zeki, 1989; Fitzpatrick et al., 1994; Schmolesky et al., 1998; Sillito et al., 2006; Briggs and Usrey, 2007, 2009). These we saw in decreasing proportion (75% and 50% respectively). It is particularly cogent to note that a component of the signal from the motion processing system is introduced into a significant proportion of the parvocellular stream at this level. Even for a single retinotopic locus the conduction time in the magnocellular system enables a signal to be reflected from MT via V1 to influence the LGN local circuitry response to the parvocellular input before the parvocellular response to the same signal has arrived (Raiguel et al., 1989; Schmolesky et al., 1998; Hupe et al., 2001; Pascual-Leone and Walsh, 2001; Sillito et al., 2006; Briggs and Usrey, 2007). Although it has been suggested that convergence in the parvo system at the level of V1 can drive shorter latency responses than the magno system (Maunsell et al., 1999) the direct evidence for the timing of magno feedback effects from V1 to LGN (Briggs and Usrey, 2007, 2011) argues for faster magno driven feedback effects as do other recent studies of responses at higher levels (Laycock et al., 2007, 2008). Moreover for moving stimuli and bipartite stimuli the latency issue is not necessarily a significant factor (Sillito et al., 2006) and it is perhaps better to consider that MT, V1 and the LGN are simultaneously involved in the ongoing representation of the stimulus (Hupe et al., 1998; Sillito et al., 2006; Gilbert and Sigman, 2007). Whilst the fact that we observed effects on the responses of LGN cells to stationary flashing stimuli may seem surprising it really only indicates that the response of an LGN cell to a flashing stimulus is different when there is an adjacent moving stimulus and that this follows at least in part from feedback from MT driven by the moving stimulus. That this difference depended on the direction of stimulus motion underlines the way feedback from MT may highlight salient differences in the neural representation of stimulus combinations. This provides support for the presence of the hypothesized reentrant mechanisms for the spatial distortions of stationary objects elicited by nearby moving stimuli (Nishida and Johnston, 1999; Whitney and Cavanagh, 2000). Similarly it is appropriate to note that the introduction of the scale of motion integration carried out in MT to the responses of an LGN cell to a stimulus over its receptive field has interesting implications for how we conceive and see the solution to the aperture problem (Marr, 1982; Pack and Born, 2001).
